# Establishment and validation of a nomogram containing cytokeratin fragment antigen 21-1 for the differential diagnosis of intrahepatic cholangiocarcinoma and hepatocellular carcinoma

**DOI:** 10.3389/fonc.2024.1404799

**Published:** 2024-06-28

**Authors:** Yuan-Yuan Liu, Yue-Yue Li, Yong-Shuai Liu, Zong-Li Zhang, Yan-Jing Gao

**Affiliations:** ^1^ Department of Gastroenterology, Qilu Hospital of Shandong University, Jinan, China; ^2^ Department of General Surgery, Qilu Hospital of Shandong University, Jinan, China

**Keywords:** CYFRA21-1, intrahepatic cholangiocarcinoma, hepatocellular carcinoma, primary liver carcinoma, serological biomarker, nomogram

## Abstract

**Background:**

Our study aimed to develop a nomogram incorporating cytokeratin fragment antigen 21–1 (CYFRA21–1) to assist in differentiating between patients with intrahepatic cholangiocarcinoma (ICC) and hepatocellular carcinoma (HCC).

**Methods:**

A total of 487 patients who were diagnosed with ICC and HCC at Qilu Hospital of Shandong University were included in this study. The patients were divided into a training cohort and a validation cohort based on whether the data collection was retrospective or prospective. Univariate and multivariate analyses were employed to select variables for the nomogram. The discrimination and calibration of the nomogram were evaluated using the area under the receiver operating characteristic curve (AUC) and calibration plots. Decision curve analysis (DCA) was used to assess the nomogram’s net benefits at various threshold probabilities.

**Results:**

Six variables, including CYFRA21–1, were incorporated to establish the nomogram. Its satisfactory discriminative ability was indicated by the AUC (0.972 for the training cohort, 0.994 for the validation cohort), sensitivity, specificity, positive predictive value (PPV), and negative predictive value (NPV) values. The Hosmer–Lemeshow test and the calibration plots demonstrated favorable consistency between the nomogram predictions and the actual observations. Moreover, DCA revealed the clinical utility and superior discriminative ability of the nomogram compared to the model without CYFRA21–1 and the model consisting of the logarithm of alpha-fetoprotein (Log AFP) and the logarithm of carbohydrate antigen 19–9 (Log CA19–9). Additionally, the AUC values suggested that the discriminative ability of Log CYFRA21–1 was greater than that of the other variables used as diagnostic biomarkers.

**Conclusions:**

This study developed and validated a nomogram including CYFRA21–1, which can aid clinicians in the differential diagnosis of ICC and HCC patients.

## 1 Introduction

Primary liver carcinoma (PLC) represents a significant global public health issue (1) and encompasses three primary histological subtypes: hepatocellular carcinoma (HCC), intrahepatic cholangiocarcinoma (ICC), and mixed hepatocellular cholangiocarcinoma (2). Although the incidence of ICC is relatively low compared to that of HCC, recent studies indicate that the incidence of ICC is gradually increasing (3). ICC and HCC differ in etiology, biology, and carcinogenic mechanism, which has been confirmed by previous studies (4–6). Thus, treatment and prognosis substantially differ (7–9). The differential diagnosis of patients with ICC and HCC remains a research focus, as effective treatment strategies depend on accurate and early differentiation.

Postoperative pathological biopsy is the gold standard for distinguishing HCC from ICC, but it is not feasible for patients with surgical contraindications. Therefore, simpler and more accurate diagnostic methods are urgently needed to facilitate early differential diagnosis and meet the diagnostic needs of patients with contraindications. While imaging technologies such as CT and MRI are prominent in differentiating HCC from ICC (7, 10–14), their limitations and dependence on technicians’ interpretation skills are notable. Ultrasound examinations also fail to provide satisfactory accuracy. Likewise, serological markers such as carbohydrate antigen 19–9 (CA19–9), alpha-fetoprotein (AFP), and inflammatory indices demonstrate limited distinguishing capabilities (15–17). Consequently, the search for more reliable diagnostic tools continues.

Cytokeratin fragment antigen 21–1 (CYFRA21–1), a fragment of cytokeratin 19, is a sensitive marker predominantly used for detecting non-small cell lung cancer (NSCLC) (18). Recent studies have shown that it is also specifically released in the serum of patients with liver and biliary diseases, particularly cholangiocarcinoma, and has attracted increasing amounts of recent attention (19, 20). Thus, the serum level of CYFRA21–1 shows promise as a marker for differentiating between ICC and HCC.

A nomogram is a predictive tool that creates simple charts based on a statistical model and is increasingly utilized to aid in clinical decision-making. The aim of this study was to develop and validate an accurate nomogram using clinical indicators obtained at hospital admission, enabling safer, simpler, and more cost-effective identification of ICC and HCC patients in the early stages of the disease, thereby facilitating individual clinical decision-making.

## 2 Materials and methods

### 2.1 Patients

This retrospective study included a total of 365 patients with pathologically confirmed diagnoses of ICC and HCC from January 2016 to April 2022 at Qilu Hospital of Shandong University; these patients composed the training cohort. The inclusion and exclusion criteria were as follows:

Inclusion criteria:

1. Patients were diagnosed with ICC or HCC based on pathological examination.
2. Age ≥ 18 years.
3. Availability of complete clinical information.
Exclusion criteria:
1. Patients with mixed tumors confirmed histopathologically.
2. Individuals with other malignancy types.
3. Patients who had undergone previous surgical treatment.
4. Those who had received radiation, chemotherapy, or antitumor drug treatment before examination were excluded.
5. Patients with a history of PLCs where the current diagnosis is a recurrence.

From April 2022 to March 2023, 122 patients who underwent partial hepatectomy for pathologically confirmed ICC and HCC and met identical inclusion and exclusion criteria were prospectively enrolled as the validation cohort. The flowchart of the study population selection process is shown in **Supplementary Figure S1**.

All procedures involving human participants adhered to the Helsinki Declaration and its later amendments. The protocols received approval from the Research Ethics Committee of Qilu Hospital of Shandong University (Approval Number: KYLL-2021–275). The study design and procedures are detailed in the study protocol (ClinicalTrials.gov: NCT05327907). Informed consent was waived for the training set due to the retrospective nature of the analysis.

### 2.2 Clinicopathologic variables

The demographic variables of the patients, including sex, age, jaundice status, smoking status, alcohol consumption status, and hepatitis history, were collected. Clinical indicators included cytokeratin fragment antigen 21–1 (CYFRA21–1), carbohydrate antigen 19–9 (CA19–9), alpha-fetoprotein (AFP), carbohydrate antigen 125 (CA125), carcinoembryonic antigen (CEA), alanine transaminase (ALT), aspartate transaminase (AST), alkaline phosphatase (ALP), albumin (ALB), total bilirubin (TBIL), direct bilirubin (DBIL), sialic acid (SA), lactate dehydrogenase (LDH), prothrombin time (PT), fibrinogen (FIB), D-dimer (D-D), white blood cell (WBC), neutrophil (NEU), lymphocyte (LYM), monocyte (MON), red blood cell (RBC), platelet (PLT), and hemoglobin (HGB) levels. These tests were analyzed before scheduled surgery at the Department of Laboratory Medicine of Qilu Hospital of Shandong University.

### 2.3 Statistical analysis

Numerical variables are presented as the means with standard deviations (SD) or medians with interquartile ranges (IQR). Student’s t-test or the Mann−Whitney test was applied for variable comparisons, as appropriate. Categorical variables are expressed as frequencies and were compared using Pearson’s χ2 test. Variables with skewed distributions, such as CYFRA21–1, CA19–9, CA125, AFP, CEA, ALP, SA, LDH, FIB, and D-dimer, underwent a logarithmic transformation.

Multivariate logistic regression analysis identified independent differential factors for ICC and HCC. The training cohort was subjected to stepwise regression based on the Akaike information criterion as a stopping rule. A nomogram was developed from these independent factors and validated in the validation cohort. The accuracy of the nomogram and its comparative discriminative performance against other models were evaluated using receiver operating characteristic (ROC) curves and area under the curve (AUC). Model consistency was assessed using calibration curves and the Hosmer–Lemeshow test. Decision curve analysis was used to quantify the net benefit of various threshold probabilities and assess the clinical utility of the nomogram and other models.

Statistical tests were two-tailed, with P < 0.05 indicating statistical significance. Analyses were conducted using R version 4.2.2 (http://www.R-project.org) and SPSS version 26.0 (IBM Corp., Armonk, NY, USA).

## 3 Results

### 3.1 Clinicopathologic characteristics of patients

During the study period, a total of 487 patients who underwent hepatectomy for primary hepatic carcinoma and met the inclusion criteria were included. The training cohort comprised 365 patients (279 with HCC and 86 with ICC), while the validation cohort consisted of 122 patients (87 with HCC and 35 with ICC). The demographics and clinicopathological variables of the patients in the training and validation cohorts are presented in **Supplementary Table S1**, and no significant differences were detected between the two cohorts. Additionally, the baseline clinicopathological data were compared between ICC patients and HCC patients in the training cohort, and the results are detailed in **Table 1**.

**Table 1:** Characteristics of patients in HCC and ICC in the training cohort.

Variables	Total (n = 365)	HCC (n = 279)	ICC (n = 86)	P value
Gender				< 0.001
Female	89 (24)	42 (15)	47 (55)	
Male	276 (76)	237 (85)	39 (45)	
Age (years)	58.93 ± 10.78	57.32 ± 10.55	64.14 ± 9.91	< 0.001
Jaundice				< 0.001
No	341 (93)	275 (99)	66 (77)	
Yes	24 (7)	4 (1)	20 (23)	
History of smoking				< 0.001
Negative	201 (55)	137 (49)	64 (74)	
Positive	164 (45)	142 (51)	22 (26)	
History of drinking				< 0.001
Negative	250 (68)	177 (63)	73 (85)	
Positive	115 (32)	102 (37)	13 (15)	
Hepatitis				< 0.001
Negative	130 (36)	55 (20)	75 (87)	
Positive	235 (64)	224 (80)	11 (13)	
CYFRA21–1 (ng/ml)	2.66 (1.81, 3.85)	2.31 (1.69, 3.1)	5.66 (3.38, 10.7)	< 0.001
CA19-9 (IU/ml)	20 (10.84, 50.61)	15.6 (9.75, 31.6)	156 (25.43, 542.82)	< 0.001
CA125 (U/ml)	14.12 (8.85, 29.1)	12.2 (8.45, 19.21)	30.92 (13.18, 105.33)	< 0.001
AFP (ng/ml)	7.24 (2.87, 164)	19.26 (3.5, 369.89)	2.92 (2.22, 5.7)	< 0.001
CEA (ng/ml)	2.51 (1.66, 4.27)	2.38 (1.54, 3.68)	3.91 (2.07, 11.73)	< 0.001
ALT (U/L)	28 (19, 46)	27 (20, 43.5)	31 (19, 83)	0.158
AST (U/L)	32 (23, 46)	31 (23, 42)	33.5 (23.25, 71.5)	0.154
ALP (U/L)	94 (72, 132)	88 (69, 111)	173 (104, 361.75)	< 0.001
ALB (g/L)	41.93 ± 6.59	42.41 ± 5.97	40.39 ± 8.14	0.035
TBIL (μmol/L)	14.5 (9.9, 21.9)	14.3 (10.2, 19.1)	15.1 (8.85, 113.33)	0.064
DBIL (μmol/L)	5.1 (3.6, 7.8)	5 (3.7, 7.2)	5.7 (3.2, 73.58)	0.054
SA (mg/dL)	55.7 (48.9, 65.3)	53.8 (47.05, 62.35)	64.9 (59.52, 74.93)	< 0.001
LDH (U/L)	212 (183, 254)	207 (181, 242)	232 (192.25, 282.75)	< 0.001
WBC (10^9^/L)	5.71 ± 2.4	5.23 ± 1.8	7.26 ± 3.3	< 0.001
NEU (10^9^/L)	3.62 ± 2.12	3.2 ± 1.49	5 ± 3.07	< 0.001
LYM (10^9^/L)	1.47 ± 0.55	1.45 ± 0.55	1.56 ± 0.55	0.091
MON (10^9^/L)	0.78 ± 5.54	0.44 ± 0.2	1.86 ± 11.4	0.251
RBC (10^12^/L)	5.55 ± 21.66	4.49 ± 0.72	8.98 ± 44.63	0.354
HGB (g/L)	135.91 ± 22.79	139.11 ± 22.98	125.53 ± 18.83	< 0.001
PLT (10^9^/L)	187.83 ± 83.54	171.04 ± 74.75	242.3 ± 87.67	< 0.001
PT (s)	13.89 ± 16.11	14.42 ± 18.38	12.17 ± 1.65	0.044
FIB (g/L)	2.96 (2.34, 3.7)	2.76 (2.23, 3.46)	3.61 (3.08, 4.44)	< 0.001
D-D (μg/ml)	0.19 (0.1, 0.51)	0.16 (0.09, 0.34)	0.44 (0.19, 0.77)	< 0.001

Numerical variables were presented as mean ± standard deviation (SD) or median with interquartile range (IQR).

HCC, hepatocellular carcinoma; ICC, intrahepatic cholangiocarcinoma; Hepatitis, history of hepatitis; CYFRA21–1, cytokeratin fragment antigen 21–1; CA19–9, carbohydrate antigen 19–9; CA125, carbohydrate antigen 125; AFP, alpha-fetoprotein; CEA, carcinoembryonic antigen; ALT, alanine transaminase; AST, aspartate transaminase; ALP, alkaline phosphatase; ALB, albumin; TBIL, total bilirubin; DBIL, direct bilirubin; SA, sialic acid; LDH, lactate dehydrogenase; WBC, white blood cell; NEU, neutrophil; LYM, lymphocyte; MON, monocyte; RBC, red blood cell; PLT, platelet; HGB, hemoglobin; PT, prothrombin time; FIB, fibrinogen; D-D, D-Dimer.

### 3.2 Univariate and multivariate analysis of differential factors between ICC and HCC

**Figure 1:**
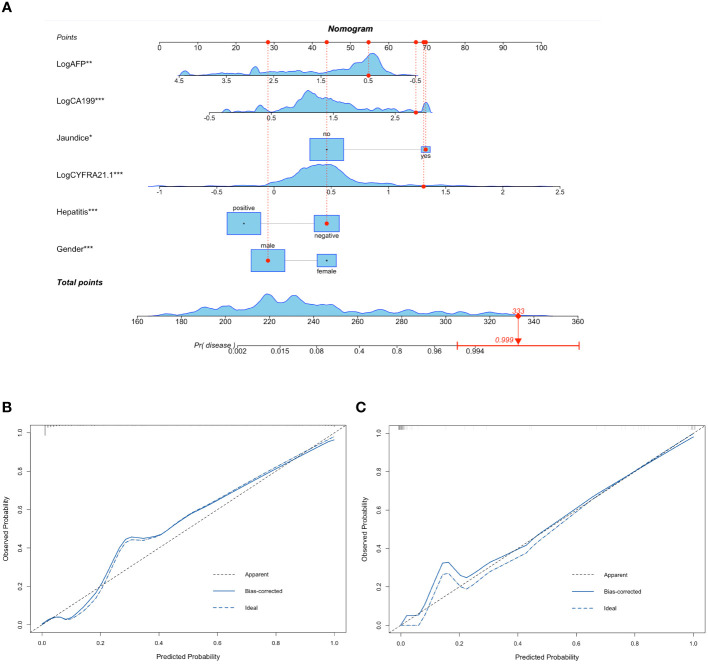
Nomogram containing CYFRA21–1 for differentiating ICC and HCC and calibration plots of nomogram. Six variables including Gender, Jaundice, Hepatitis, Log CYFRA21–1, Log CA19–9 and Log AFP were selected to establish the nomogram. For example, a 71-year-old male patient with jaundice and no history of hepatitis, CA19–9 of 680.3IU/ml, AFP of 3.11ng/ml, CYFRA21–1 of 20.30ng/ml had a 99.9% probability of diagnosing ICC **(A)**. The calibration curves of the nomogram in the training **(B)** and validation **(C)** cohorts. The calibration curves of the nomogram showed good consistency between the predicted probability of ICC diagnosis and the actual probability. HCC, hepatocellular carcinoma; ICC, intrahepatic cholangiocarcinoma; Log CYFRA21–1, logarithm of cytokeratin fragment antigen 21–1; Log CA19–9, logarithm of carbohydrate antigen 19–9; Log AFP, logarithm of alpha-fetoprotein. * represented P value < 0.05 between ICC group and HCC group; ** represented P value < 0.01 between ICC group and HCC group; *** represented P value ≤ 0.001 between ICC group and HCC group.

**Table 2** summarizes the results of the univariate and multivariate logistic analyses. Twenty-two candidate variables were initially identified by univariate analysis as significantly different between ICC and HCC patients in the training cohort. Following backward stepwise selection and multivariate logistic regression, six independent indicators for ICC (sex, jaundice status, hepatitis status, logarithm of CYFRA21–1 (Log CYFRA21–1), logarithm of CA19–9 (Log CA19–9) and logarithm of AFP (Log AFP)) were selected and integrated into the nomogram. We calculated the total number of points on the vertical line from each variable to the point axis to calculate the probability of diagnosing ICC (**Figure 1A**). The P values of the Hosmer and Lemeshow tests in the training and validation cohorts were 0.0824 and 0.8486, respectively, indicating non-significance. The calibration curves for the nomogram displayed good consistency between the predicted and actual probabilities of ICC diagnosis in the training and validation cohorts (**Figures 1B, C**).

**Table 2:** Univariate and multivariate logistic regression analysis of ICC presence based on preoperative data in training cohort.

	Univariate logistic regression analysis		multivariate logistic regression analysis	
term	Odds ratio (95% CI)	p	Odds ratio (95% CI)	p
Gender male	0.147 [0.085, 0.250]	<0.001	0.20 [0.07, 0.51]	0.001
Age (years)	1.067 [1.040, 1.095]	<0.001		
Jaundice yes	20.833 [7.584, 73.462]	<0.001	15.20 [2.03, 149.98]	0.013
Smoking history positive	0.332 [0.190, 0.560]	<0.001		
Drinking history positive	0.309 [0.157, 0.568]	<0.001		
Hepatitis positive	0.036 [0.017, 0.070]	<0.001	0.10 [0.04, 0.27]	<0.001
Log CYFRA21–1 (ng/ml)	96.633 [32.429, 338.361]	<0.001	23.23 [6.77, 112.29]	<0.001
Log CA19–9 (IU/ml)	7.281 [4.598, 12.086]	<0.001	5.49 [2.63, 12.76]	<0.001
Log CA125 (U/ml)	5.676 [3.378, 9.971]	<0.001		
Log AFP (ng/ml)	0.230 [0.136, 0.355]	<0.001	0.27 [0.11, 0.55]	0.002
Log CEA (ng/ml)	8.344 [4.219, 17.984]	<0.001		
Log ALP (U/L)	117.620 [36.722, 437.379]	<0.001		
ALB (g/L)	0.949 [0.910, 0.988]	0.013		
Log SA (mg/dL)	8472.251[585.061,150313.450]	<0.001		
Log LDH (U/L)	16.250 [2.546, 107.023]	0.003		
WBC (10^9^/L)	1.468 [1.299, 1.674]	<0.001		
NEU (10^9^/L)	1.562 [1.356, 1.820]	<0.001		
HGB (g/L)	0.975 [0.964, 0.986]	<0.001		
PLT (10^9^/L)	1.011 [1.008, 1.015]	<0.001		
PT (s)	0.901 [0.749, 0.998]	<0.001		
Log FIB (g/L)	148.362 [26.967, 967.379]	0.001		
Log D-D (μg/ml)	4.766 [2.807, 8.351]	<0.001		

ICC, intrahepatic cholangiocarcinoma; Hepatitis, history of hepatitis; CYFRA21–1, cytokeratin fragment antigen 21–1; Log CA19–9, logarithm of carbohydrate antigen 19–9; Log CA125, logarithm of carbohydrate antigen 125; Log AFP, logarithm of alpha-fetoprotein; Log CEA, logarithm of carcinoembryonic antigen; Log ALP, logarithm of alkaline phosphatase; ALB, albumin; Log SA, logarithm of sialic acid; Log LDH, logarithm of lactate dehydrogenase; WBC, white blood cell; NEU, neutrophil; HGB, hemoglobin; PLT, platelet; PT, prothrombin time; Log FIB, logarithm of fibrinogen; Log D-D, logarithm of D-Dimer.

### 3.3 Development and validation of a nomogram for ICC differential diagnosis

**Figure 2:**
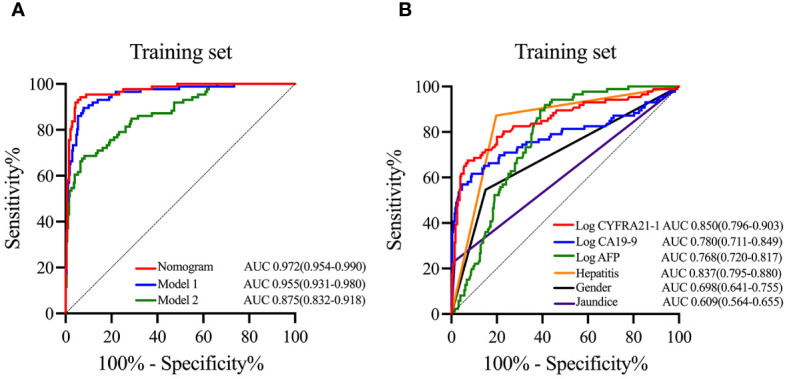
ROC curves of the nomogram, models and variables in the training cohort. In the training cohort, ROC curves of the nomogram, Model 1 and Model 2 **(A)**, and ROC curves and AUC of six variables including in the nomogram **(B)**. ROC curves, receiver operating characteristic curves; AUC, area under the curve; Log CYFRA21–1, logarithm of cytokeratin fragment antigen 21–1; Log CA19–9, logarithm of carbohydrate antigen 19–9; Log AFP, logarithm of alpha-fetoprotein.

**Figure 3:**
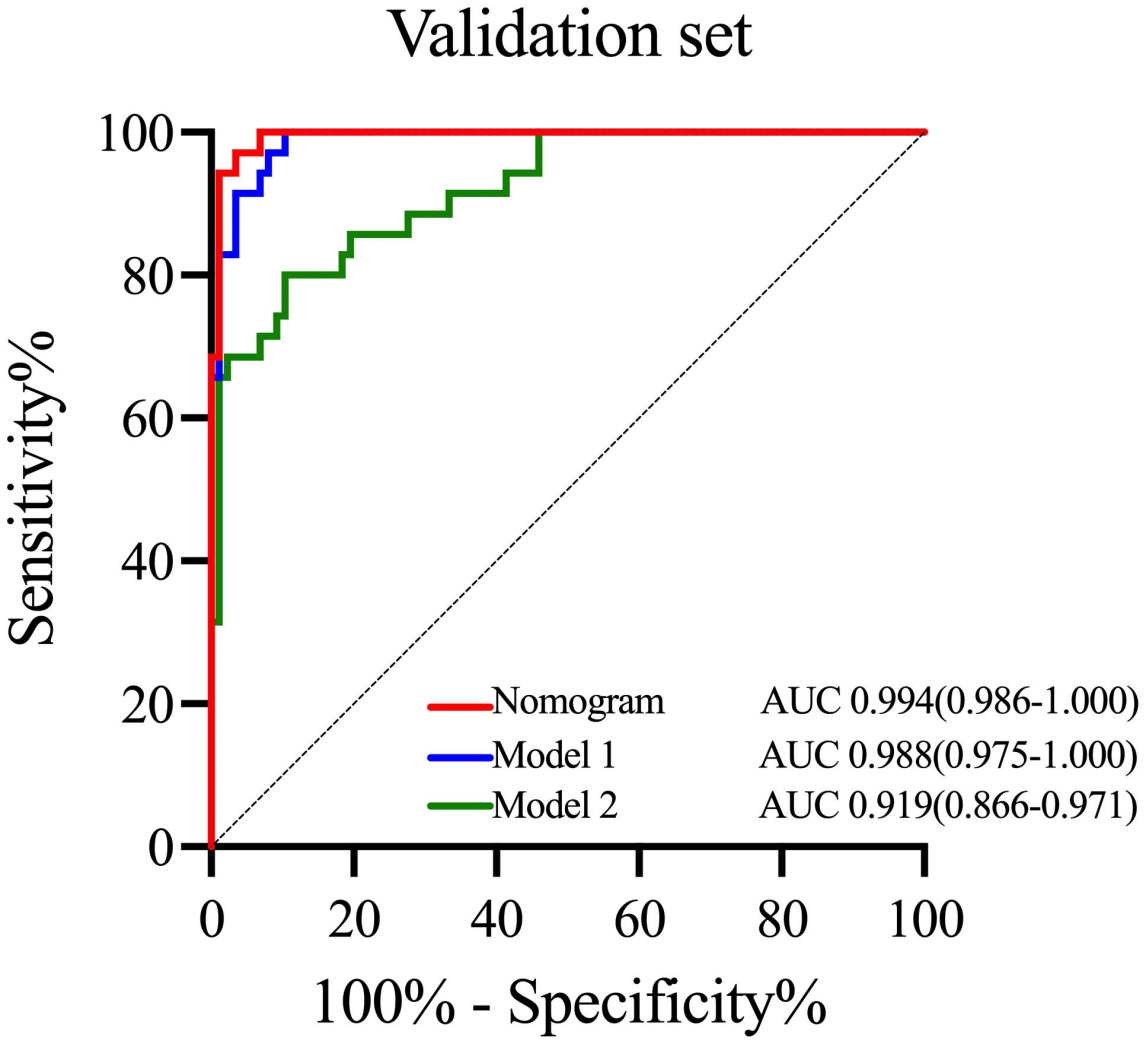
ROC curves of the nomogram and other models in the validation cohort. ROC curves of the nomogram, Model 1 and Model 2 in the validation cohort. ROC curves, receiver operating characteristic curves; AUC, area under the curve.

**Figure 4:**
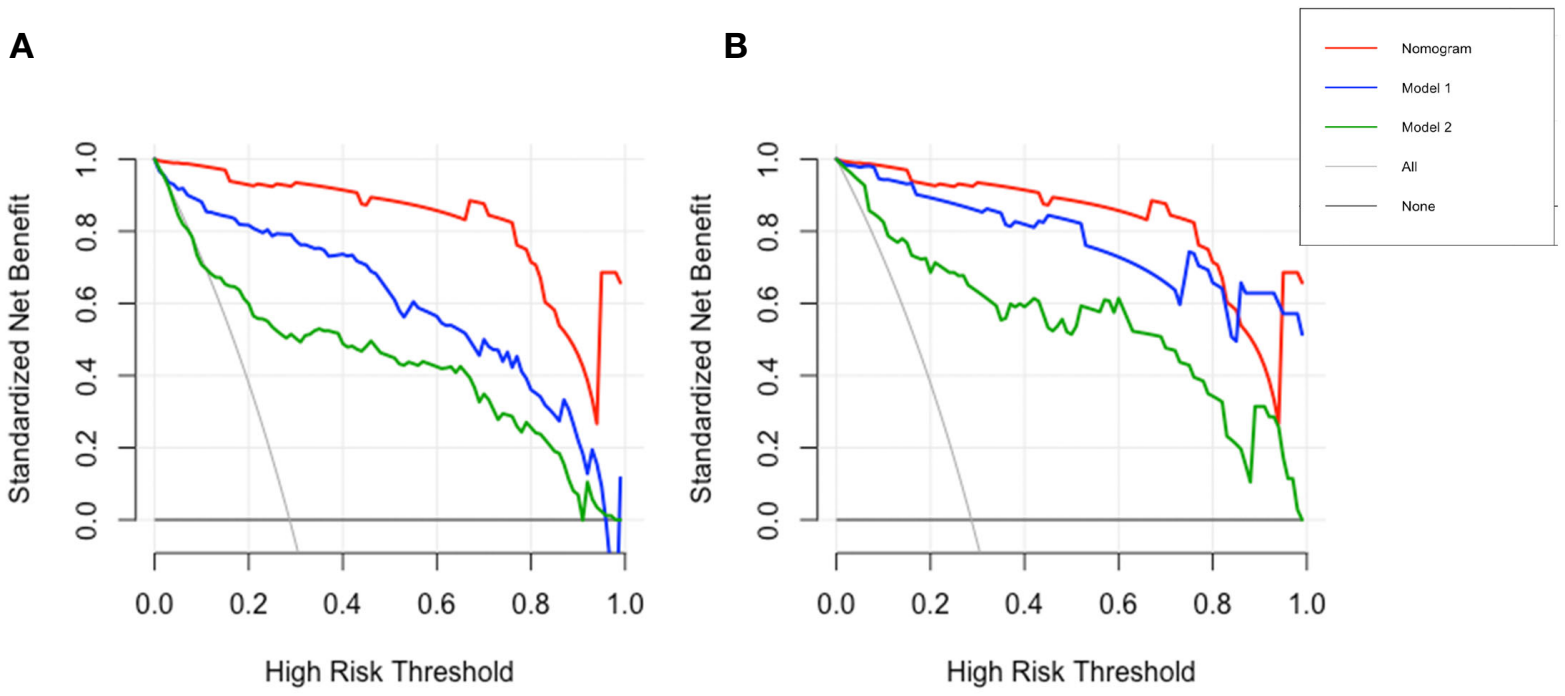
DCA of nomogram and models. DCA of the nomogram, Model 1 and Model 2 in the training **(A)** and validation **(B)** cohorts, the x- and y-axes respectively show the risk threshold probability and net benefit. DCA, decision curve analysis.

To emphasize the significance of CYFRA21–1, Model 1 was constructed, which included sex, jaundice, hepatitis, Log CA19–9 and Log AFP. Additionally, Model 2 was established by incorporating Log AFP and Log CA19–9, which are commonly used in clinical practice. The performance of the nomogram and the significance of CYFRA21–1 were evaluated from various perspectives. In the training cohort, the AIC values of the nomogram were lower than those of Model 1 and Model 2 (AIC of nomogram = 141.8, AIC of Model 1 = 170.52, AIC of Model 2 = 255.02). The nomogram exhibited superior differentiation of ICC and HCC, with an AUC of 0.972 (95% CI, 0.954–0.990), compared to Model 1 (AUC = 0.955, 95% CI, 0.931–0.980) and Model 2 (AUC = 0.875, 95% CI, 0.832–0.918), as depicted in **Figure 2A**. There were statistically significant differences in the area under the curve (AUC) between the nomogram and models 1 (P value = 0.046) and 2 (P value < 0.001). Additionally, the individual ROC curves for the six variables in the nomogram revealed that the area under the curve (AUC) for Log CYFRA21–1 (AUC = 0.850, 95% CI: 0.796–0.903) was greater than those for the other five variables, including Log CA19–9 (AUC = 0.780, 95% CI: 0.711–0.849) and Log AFP (AUC = 0.768, 95% CI: 0.720–0.817) (**Figure 2B**), demonstrating that CYFRA21–1 plays a significant role in the differential diagnosis of ICC and HCC. In the validation cohort, the nomogram also had an AUC of 0.994 (95% CI, 0.986–1.000) for differentiating ICC from HCC compared to Model 1 (AUC = 0.988, 95% CI: 0.975–1.000) and Model 2 (AUC = 0.919, 95% CI: 0.866–0.971), as shown in **Figure 3**. The sensitivity, specificity, positive predictive value (PPV), and negative predictive value (NPV) of the nomogram, Model 1, and Model 2 in the training and validation cohorts were compared and are illustrated in **Table 3**. The nomogram was superior to Model 1 and Model 2. The sensitivities were 94.2%, 89.5%, and 68.6%; the specificities were 93.5%, 92.1%, and 91.8%; the PPV were 81.8%, 77.8%, and 72%; and the NPV were 98.1%, 96.6%, and 90.5% for the nomogram, Model 1 and Model 2, respectively, in the training set. In the validation set, the sensitivity was 97.1%, 100%, and 80%; the specificity was 96.6%, 89.7%, and 89.7%; the PPV was 91.9%, 79.5%, and 75.7%; and the NPV was 98.8%, 100%, and 91.8% for Model 1 and Model 2, respectively. DCA revealed that utilizing the nomogram for distinguishing ICC from HCC provided more benefits than Models 1 and 2 (**Figures 4A, B**).

**Table 3:** Diagnostic efficacy of different methods.

	AUC (95% CI)	Sensitivity (%)	Specificity (%)	PPV (%)	NPV (%)
Training cohort					
Nomogram	0.972 (0.954–0.990)	94.2	93.5	81.8	98.1
Model 1	0.955 (0.931–0.980)	89.5	92.1	77.8	96.6
Model 2	0.875 (0.832–0.918)	68.6	91.8	72	90.5
Validation cohort					
Nomogram	0.994 (0.986–1.000)	97.1	96.6	91.9	98.8
Model 1	0.988 (0.975–1.000)	100	89.7	79.5	100
Model 2	0.919 (0.866–0.971)	80	89.7	75.7	91.8

AUC, Area under the receiver operating characteristic; CI, confidence interval; PPV, Positive predictive value; NPV, Negative predictive value.

## 4 Discussion

The epidemiology, risk factors, genetics, and epigenetics of ICC and HCC vary significantly (9), accompanied by notable differences in cellular metabolism, leading to distinct treatment approaches and prognoses for each (4, 21–23). In summary, precise and accurate differential diagnosis is imperative in navigating the complexities of these distinct liver cancers.

Our study combined CYFRA21–1 with traditional differential diagnostic indicators (sex, jaundice, hepatitis, Log AFP, and Log CA19–9) to increase the accuracy and specificity of differentiating ICC from HCC and developed a nomogram that achieved greater benefit than did previous models, potentially aiding in therapeutic decision-making.

Female sex was found to be positively associated with ICC, and a history of hepatitis was negatively associated with ICC, which aligns with the findings of previous studies (7, 24). Jaundice is positively associated with ICC, which can be attributed to the fact that the location of the ICC is more prone to causing biliary obstruction than the location of the HCC (25).

As CA19–9 and AFP are widely used biomarkers for diagnosing ICC and HCC, respectively, the combined use of CA19–9 and AFP levels is prevalent in distinguishing ICC from HCC in clinical practice (7, 16, 20), and we constructed this model (Model 2) in our study. Compared to Model 2 (AUC = 0.875), our nomogram had a greater AUC of 0.972 and was shown to provide superior discriminative ability between ICC and HCC.

Researchers have previously conducted similar studies. Wang et al. utilized six easily obtainable parameters to develop a nomogram for distinguishing between ICC and HCC (26). However, their nomogram presented the indicators as categorical variables, whereas our study used continuous variables for a more accurate and straightforward analysis. Si et al. also constructed a nomogram using clinical indicators (27), but their study included only laboratory test indicators. Previous studies have shown significant differences in the epidemiology, risk factors and clinical presentation of ICC and HCC, and our study analyzed their value in discriminating diagnosis, with the results demonstrating that sex and the presence of jaundice symptoms are independent risk factors for differentiating between ICC and HCC. Furthermore, our study incorporated CYFRA21–1 levels and revealed that it has significant value in discriminating between ICC and HCC. The inclusion of CYFRA21–1 improved the performance of the nomogram significantly compared to that without this indicator. Additionally, the AUC of CYFRA21–1 was greater than that of the other independent risk factors selected, indicating its significance in discriminating between ICC and HCC.

Cytokeratins (CKs) are intermediate filaments found in the cytoskeleton of almost all epithelial cells. They play a crucial role in the stability of epithelial cells and many intracellular signaling cascades (28, 29). Activated proteases in malignant epithelial cells can promote CK degradation, leading to high expression of CK fragments (30). Severe chronic liver damage induces a ductular reaction (DR) composed of ductal cells and liver progenitor cells (LPCs), with CK19 being a prominent histological marker for DR (31, 32). Therefore, the expression of CK19 may be related to the diagnosis and progression of liver and biliary tract diseases. CYFRA21–1, a soluble fragment of CK19 and a useful marker for non-small cell lung cancer (NSCLC) (18), has been gaining attention for its potential role in the diagnosis and prognosis of liver and biliary tract diseases (33, 34). A previous study linked CK19 expression with the progression of ICC and demonstrated higher CYFRA21–1 serum levels in ICC patients than in those with extrahepatic adenocarcinoma (35). However, few studies have compared the serum CYFRA21–1 concentration between patients with ICC and patients with HCC. Given the predominant expression of CK19 in chronic biliary tract disease and the absence of CK19 in hepatocytes (36, 37), CYFRA21–1 levels are expected to be greater in ICC than in HCC, a hypothesis supported by our study. This study established that CYFRA21–1 is an independent risk factor for distinguishing between ICC and HCC, and the AUC of CYFRA21–1 was greater than that of Log CA19–9 and Log AFP, which indicates that CYFRA21–1 plays a significant role in the differential diagnosis of ICC and HCC.

However, this study has several limitations. The data were sourced from a single institution, highlighting the need for further validation with a larger external sample. Additionally, the relatively small sample size of this study necessitates further research with larger cohorts to ascertain the definitive impact of the serum CYFRA21–1 concentration in differentiating between ICC and HCC. Furthermore, this study identified only ICC and HCC, and further research is needed to explore whether CYFRA21–1 can play a role in differentiating ICC from other types of liver cancer, including mixed hepatocellular-cholangiocarcinoma and liver metastases.

## 5 Conclusion

In conclusion, we developed a nomogram with a superior AUC compared to that of previous models, and its predictive ability was assessed from various perspectives. Furthermore, this study underscores the clinical significance of CYFRA21–1 in differentiating between ICC and HCC patients and offers a novel approach for differential diagnosis.

## Data availability statement

The raw data supporting the conclusions of this article will be made available by the authors, without undue reservation.

## Ethics statement

The studies involving humans were approved by Shandong University Qilu Hospital Ethics Committee. The studies were conducted in accordance with the local legislation and institutional requirements. The participants provided their written informed consent to participate in this study.

## Author contributions

Y-YLiu: Data curation, Formal analysis, Validation, Visualization, Writing – original draft. Y-YLi: Project administration, Supervision, Writing – review & editing. Y-SL: Data curation, Validation, Writing – original draft. Z-LZ: Conceptualization, Investigation, Methodology, Supervision, Writing – review & editing. Y-JG: Conceptualization, Investigation, Methodology, Project administration, Supervision, Writing – review & editing.
