# Supplementary material for: Establishment and validation of a nomogram containing cytokeratin fragment antigen 21-1 for the differential diagnosis of intrahepatic cholangiocarcinoma and hepatocellular carcinoma
Source: Front Oncol. 2024 Jun 28;14:1404799. doi: 10.3389/fonc.2024.1404799 (PMC11239389; doi:10.3389/fonc.2024.1404799)
Supplement: Supplementary file 1 [file DataSheet_1.docx]

Supplementary Material

## Supplementary Figure

**
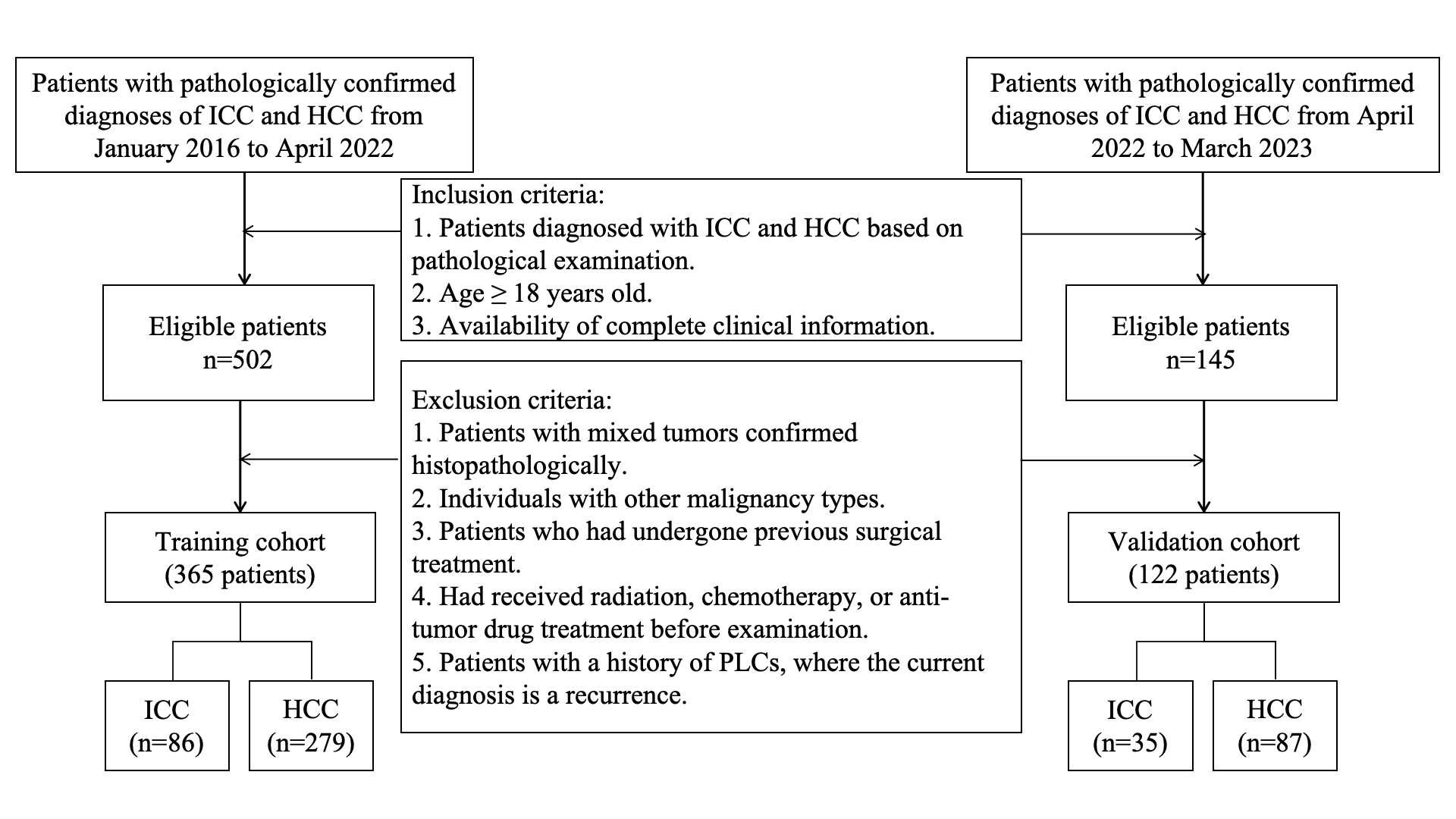
**

**Supplementary Figure 1.** Flow diagram of patient selection.

The flowchart of the study population selection process.

## Supplementary Table

**Supplementary Table 1.** Characteristics of patients in HCC and ICC.

| Variables | Total  (n = 487) | Training cohort  (n = 365) | Validation cohort  (n = 122) | P value |
| --- | --- | --- | --- | --- |
| Disease |  |  |  | 0.257 |
| HCC | 366 (75) | 279 (76) | 87 (71) |  |
| ICC | 121 (25) | 86 (24) | 35 (29) |  |
| Gender |  |  |  | 0.556 |
| Female | 122 (25) | 89 (24) | 33 (27) |  |
| Male | 365 (75) | 276 (76) | 89 (73) |  |
| Age (years) | 58.69 ± 10.77 | 58.93 ± 10.78 | 57.98 ± 10.75 | 0.398 |
| Jaundice |  |  |  | 0.510 |
| No | 457 (94) | 341 (93) | 116 (95) |  |
| Yes | 30 (6) | 24 (7) | 6 (5) |  |
| History of smoking |  |  |  | 0.977 |
| Negative | 268 (55) | 201 (55) | 67 (55) |  |
| Positive | 219 (45) | 164 (45) | 55 (45) |  |
| History of drinking |  |  |  | 0.112 |
| Negative | 324 (67) | 250 (68) | 74 (61) |  |
| Positive | 163 (33) | 115 (32) | 48 (39) |  |
| Hepatitis |  |  |  | 0.812 |
| Negative | 172 (35) | 130 (36) | 42 (34) |  |
| Positive | 315 (65) | 235 (64) | 80 (66) |  |
| CYFRA21-1 (ng/ml) | 2.71 (1.79, 3.86) | 2.66 (1.81, 3.85) | 2.78 (1.71, 3.87) | 0.962 |
| CA19-9 (IU/ml) | 18.8 (10.57, 53.08) | 20 (10.84, 50.61) | 18.62 (9.94, 60.05) | 0.839 |
| CA125 (U/ml) | 13.76 (8.68, 26.5) | 14.12 (8.85, 29.1) | 13.52 (8.36, 22.51) | 0.369 |
| AFP (ng/ml) | 6.84 (2.88, 200.74) | 7.24 (2.87, 164) | 5.58 (3.07, 249.94) | 0.837 |
| CEA (ng/ml) | 2.48 (1.6, 4.08) | 2.51 (1.66, 4.27) | 2.48 (1.48, 3.75) | 0.325 |
| ALT (U/L) | 27 (19, 46) | 28 (19, 46) | 25 (17, 42.5) | 0.110 |
| AST (U/L) | 31 (23, 44) | 32 (23, 46) | 28 (21, 43.75) | 0.078 |
| ALP (U/L) | 94 (73.5, 137.5) | 94 (72, 132) | 92 (75.25, 141) | 0.959 |
| ALB (g/L) | 41.99 ± 6.19 | 41.93 ± 6.59 | 42.15 ± 4.81 | 0.695 |
| TBIL (μmol/L) | 14 (9.9, 20.1) | 14.5 (9.9, 21.9) | 12.8 (9.93, 18.67) | 0.115 |
| DBIL (μmol/L) | 5 (3.6, 7.5) | 5.1 (3.6, 7.8) | 4.6 (3.52, 6.88) | 0.137 |
| SA (mg/dL) | 55.7 (49.2, 65.85) | 55.7 (48.9, 65.3) | 55.8 (50.6, 68.57) | 0.385 |
| LDH (U/L) | 211 (182.5, 252) | 212 (183, 254) | 210 (181, 243.75) | 0.371 |
| WBC (10^9^/L) | 5.75 ± 2.32 | 5.71 ± 2.4 | 5.9 ± 2.08 | 0.401 |
| NEU (10^9^/L) | 3.79 ± 3.78 | 3.62 ± 2.12 | 4.28 ± 6.59 | 0.282 |
| LYM (10^9^/L) | 1.5 ± 0.56 | 1.47 ± 0.55 | 1.58 ± 0.58 | 0.079 |
| MON (10^9^/L) | 0.7 ± 4.8 | 0.78 ± 5.54 | 0.47 ± 0.21 | 0.289 |
| RBC (10^12^/L) | 5.25 ± 18.75 | 5.55 ± 21.66 | 4.36 ± 0.65 | 0.294 |
| HGB (g/L) | 135.6 ± 22.75 | 135.91 ± 22.79 | 134.68 ± 22.68 | 0.607 |
| PLT (10^9^/L) | 188.9 ± 85.9 | 187.83 ± 83.54 | 192.08 ± 92.9 | 0.654 |
| PT (s) | 13.65 ± 14.68 | 13.89 ± 16.11 | 12.93 ± 9.19 | 0.418 |
| FIB (g/L) | 2.96 (2.4, 3.67) | 2.96 (2.34, 3.7) | 3 (2.48, 3.61) | 0.505 |
| D-D (μg/ml) | 0.19 (0.1, 0.52) | 0.19 (0.1, 0.51) | 0.2 (0.1, 0.53) | 0.917 |

Numerical variables were presented as mean ± standard deviation (SD) or median with interquartile range (IQR).

Abbreviations: HCC, hepatocellular carcinoma; ICC, intrahepatic cholangiocarcinoma; Hepatitis, history of hepatitis; CYFRA21-1, cytokeratin fragment antigen 21-1; CA19-9, carbohydrate antigen 19-9; CA125, carbohydrate antigen 125; AFP, alpha-fetoprotein; CEA, carcinoembryonic antigen; ALT, alanine transaminase; AST, aspartate transaminase; ALP, alkaline phosphatase; ALB, albumin; TBIL, total bilirubin; DBIL, direct bilirubin; SA, sialic acid; LDH, lactate dehydrogenase; WBC, white blood cell; NEU, neutrophil; LYM, lymphocyte; MON, monocyte; RBC, red blood cell; PLT, platelet; HGB, hemoglobin; PT, prothrombin time; FIB, fibrinogen; D-D, D-Dimer
